# Giant malignant phyllodes tumor: A case report

**DOI:** 10.1016/j.radcr.2025.04.078

**Published:** 2025-05-13

**Authors:** Daniela Kerguelen Murcia, Andrea Tenreiro, Jennifer A. Kohlnhofer, Celeste Wagner, Flavia Posleman Monetto

**Affiliations:** a John Sealy School of Medicine, University of Texas Medical Branch, Galveston, TX, USA; b Department of Radiology, University of Texas Medical Branch, Galveston, TX, USA; c Department of Pathology, University of Texas Medical Branch, Galveston, TX, USA

**Keywords:** Phyllodes tumor, Breast imaging, Breast mass, Radiology

## Abstract

Phyllodes tumor is a rare fibroepithelial breast mass that typically manifests as a painless, multinodular breast tumor. One characteristic feature of phyllodes tumors is that they tend to increase in size rapidly over time unlike fibroadenomas. On ultrasound, they typically appear as a hypoechoic solid mass that may contain cystic components. On mammography, they appear hyperdense. Definitive diagnosis requires biopsy, with histological findings of leaf-like architecture and stromal cellularity. This report presents a case of giant malignant phyllodes tumor in a 53-year-old female who underwent right mastectomy and provides an overview of its clinical presentation, diagnostic workup, radiologic imaging findings, and management.

## Introduction

Phyllodes tumor is a rare fibroepithelial breast mass that accounts for less than 1 percent of female breast tumors, and typically manifests in women aged 45-49 as a painless, multinodular tumor [1]. Fibroepithelial tumors are comprised of stromal and epithelial components and includes fibroadenomas and phyllodes tumors. Unlike fibroadenomas, phyllodes tumors tend to increase in size rapidly over time. The median size of phyllodes tumors is 4 cm, while giant phyllodes tumors are those larger than 10 cm and comprise about 20% of tumors [2,3].

Diagnostic workup for a palpable breast mass includes mammography and ultrasound. On ultrasound, phyllodes tumors typically appear as a hypoechoic solid mass containing cystic components. On mammography, they appear hyperdense [4]. However, definitive diagnosis requires biopsy, with histological findings of leaf-like architecture and stromal cellularity. Histologically, they can be categorized as benign, borderline, and malignant [5]. Here, we report a case of a giant malignant phyllodes tumor.

## Case presentation

A 53-year-old woman with no previous mammography presented to the emergency department (ED) with a large right breast mass that had been present for a year but was rapidly growing over the past 2 months. Physical exam was remarkable for a large right breast mass with visible distortion of the chest. It appeared pendulous, with significant skin stretching due to its size and weight. The breast was also tender to palpation with solid and fluctuant areas and without nipple discharge.

**Fig. 1:**
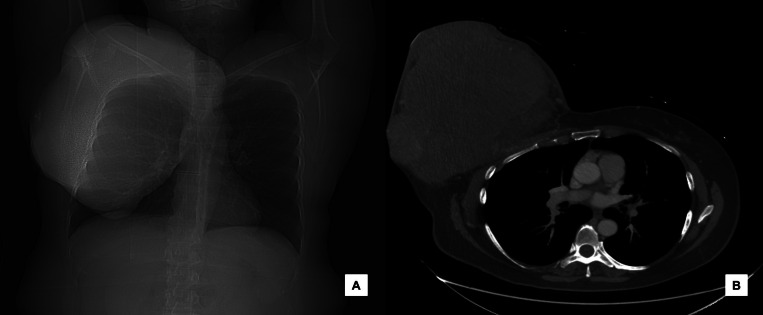
(A) Scout image of the thorax demonstrating a large mass occupying the entire right breast and (B) Axial contrast-enhanced CT image of the chest demonstrating a lobulated mass with heterogenous enhancement.

A computed tomography (CT) thorax was initially ordered in the ED, which showed a lobulated mass with heterogeneous enhancement measuring approximately 23 × 22 × 28 cm in size (AP x TV x CC) in the right breast without chest wall invasion beyond the pectoralis major muscle (Fig. 1). The patient was referred for further radiographic workup including mammography and ultrasound.

**Fig. 2:**
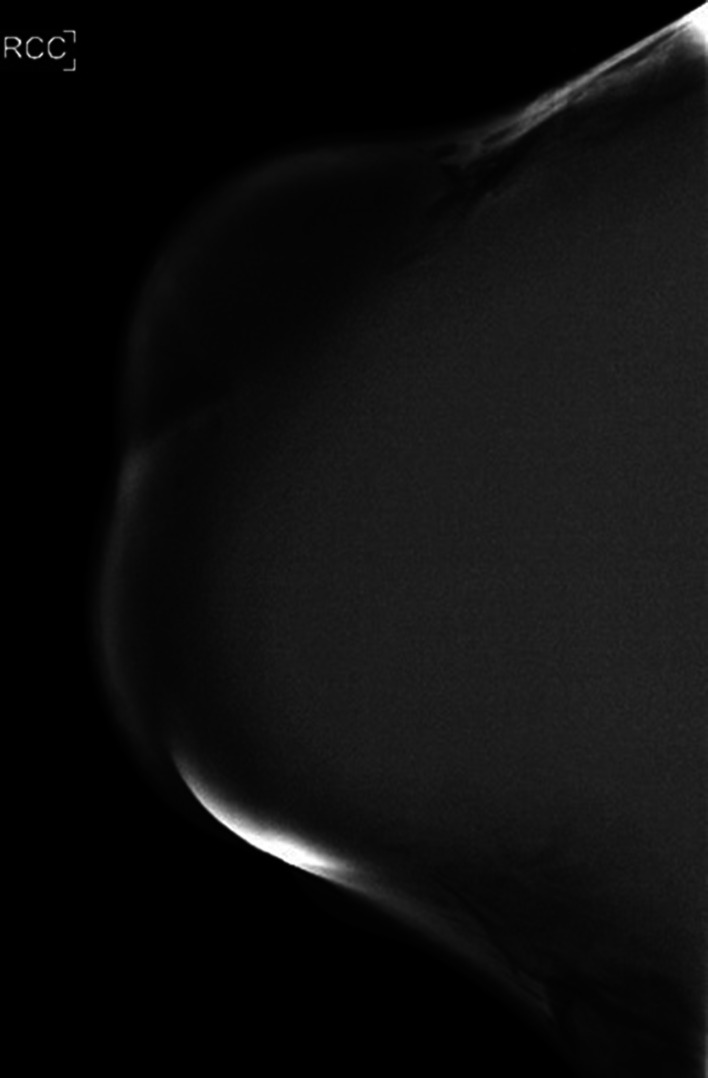
Craniocaudal 2D mammographic view demonstrating a large mass occupying the entire right breast.

Due to the extensive size of the mass, only a 2D mammographic image was acquired, as the breast could not be adequately compressed to obtain tomosynthesis images. The image demonstrated a large right mass encompassing the entire right breast (Fig. 2).

**Fig. 3:**
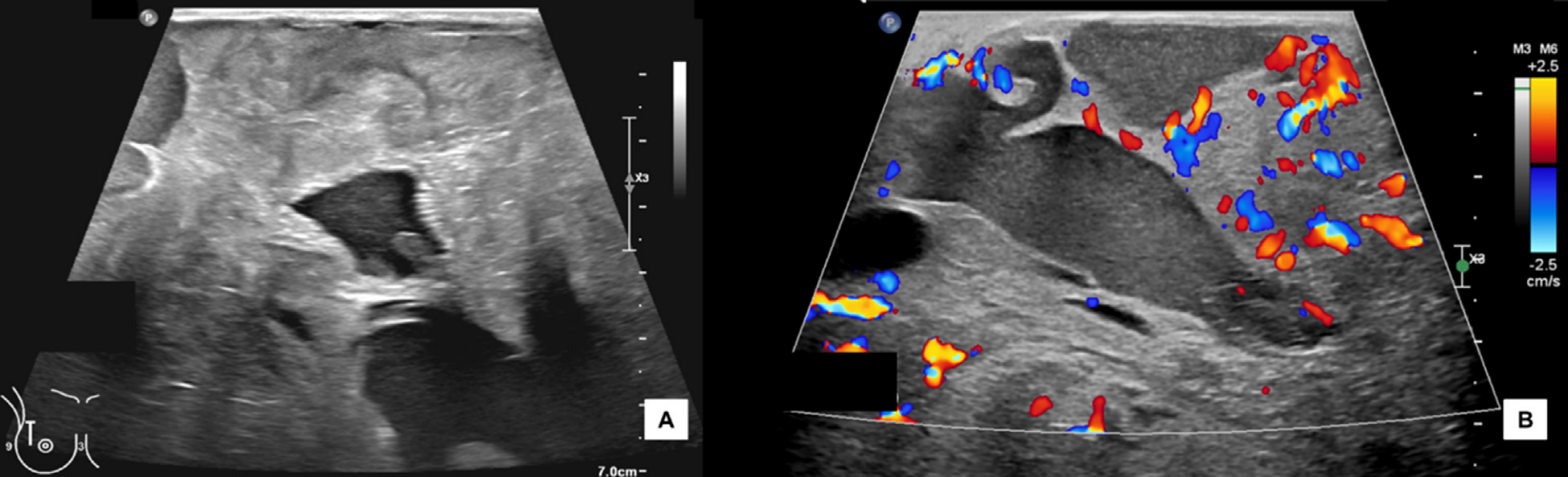
(A) Ultrasound of the right breast demonstrating the large heterogenous mass with cystic spaces in the upper outer quadrant and (B) color Doppler ultrasound showing internal vascularity in the upper inner quadrant of the mass.

On ultrasound of the right breast there was a palpable cystic and solid mass with internal vascularity involving all quadrants of the right breast and the subareolar region which was too large to accurately measure on ultrasound. However, this mass extended up to 28 cm from the nipple in the upper quadrants (Fig. 3). No axillary, supraclavicular, or internal mammary chain lymphadenopathy was seen. A representative solid area was noted in the upper outer quadrant, 8 cm from the nipple. The mass was characterized as Breast Imaging Reporting and Data System (BI-RADS) category 5, and the patient was scheduled for an ultrasound-guided biopsy.

**Fig. 4:**
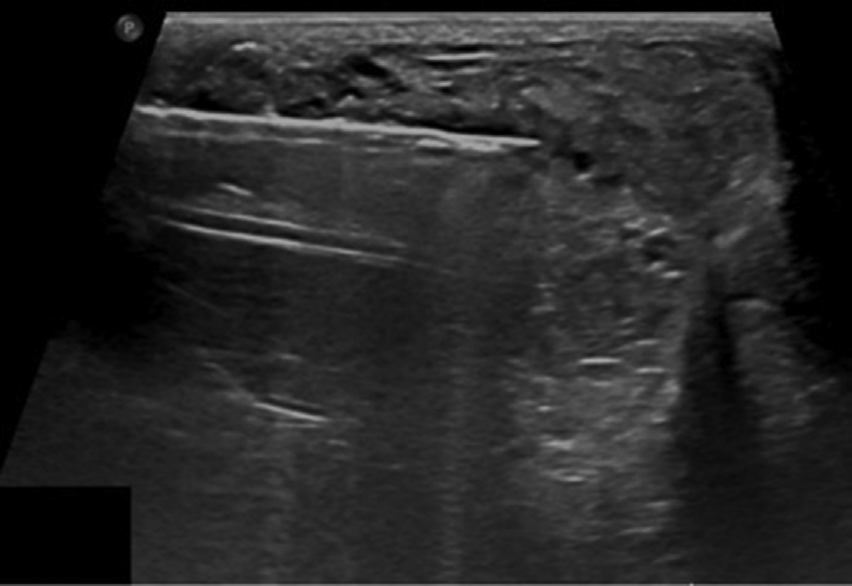
Ultrasound guided core biopsy with 14-gauge automated device of the right breast mass in the upper outer quadrant, 8 cm from the nipple.

**Fig. 5:**
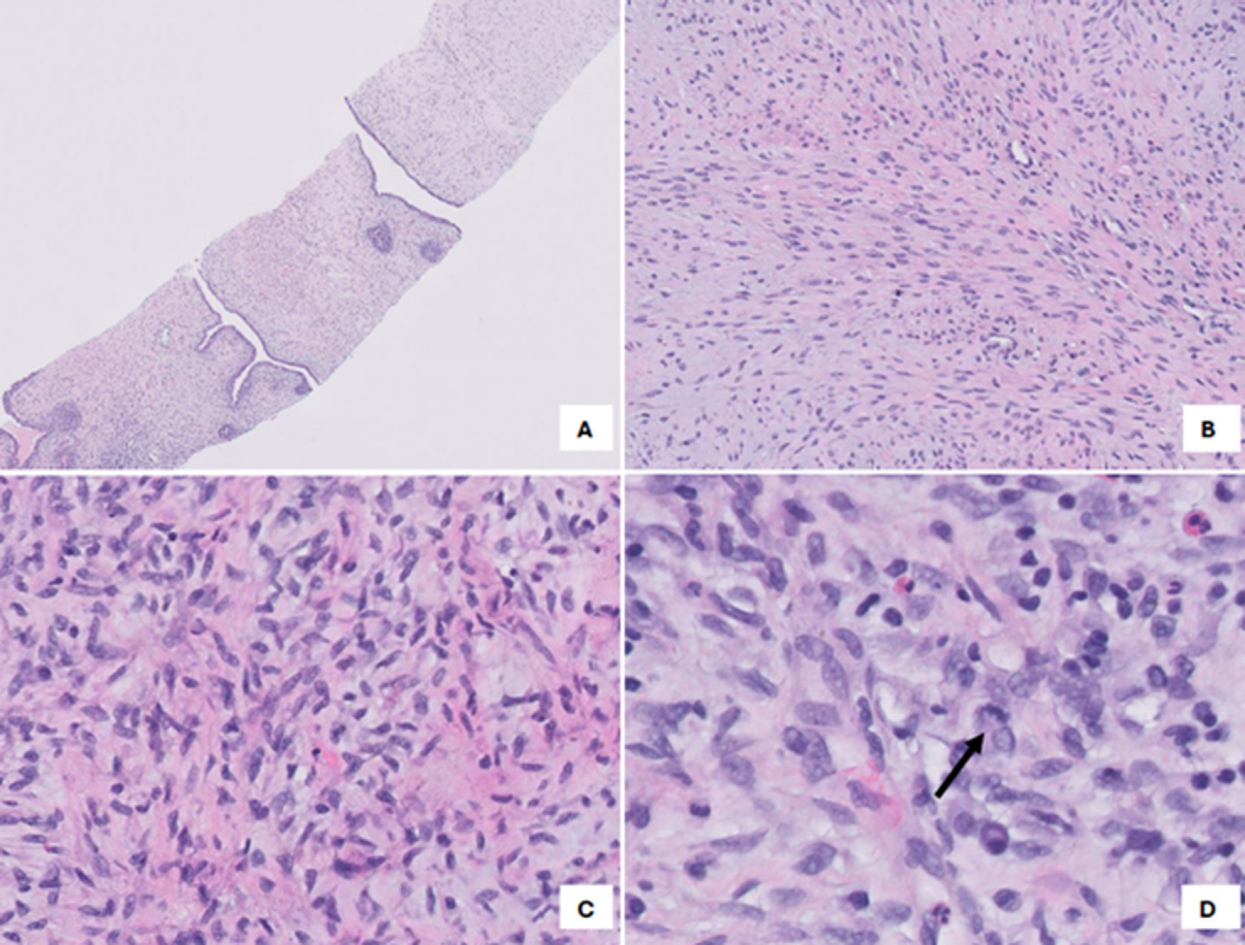
(A) Pathology slide at low magnification demonstrating a biphasic fibroepithelial lesion with a leaf-like pattern. (B) and (C) Stromal expansion with areas of stromal overgrowth. (D) At high magnification cytologic atypia and mitotic figures (arrow) are noted.

Ultrasound-guided biopsy (Fig. 4) showed a biphasic fibroepithelial lesion with a leaf-like epithelial pattern. The stromal cellularity was marked in a few areas and cells exhibited moderate atypia. Focal stromal overgrowth was also present. The mitotic count was <5 per 10 HPF. These findings were compatible with phyllodes tumor (Fig. 5). The final diagnosis was determined to be a malignant phyllodes tumor and the patient was referred to surgery and oncology.

**Fig. 6:**
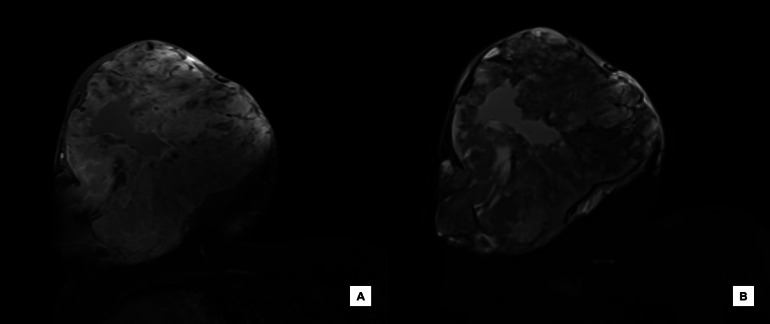
(A) Axial T1-weighted postcontrast fat saturated MRI image of the right breast demonstrating a 3.1 cm mass with avid, heterogenous enhancement and areas of intermediate signal intensity suggestive of hemorrhage and (B) Axial T2-weighted MRI image of the same mass demonstrating variable signal intensity.

Preoperatively, magnetic resonance imaging (MRI) was ordered for tumor extent assessment and preoperative planning. On MRI, the right breast multilobulated mass measured approximately 31 × 21 × 24 cm. The internal signal was mostly T1 hypointense (Fig. 6A), T2 variable (Fig. 6B), and the solid components were intensely enhanced. Areas of intermediate T1 signal intensity likely correlate with hemorrhage. The mass appeared intimately associated with the pectoralis musculature and abutted the underlying chest wall.

The patient underwent a right simple mastectomy and coverage with split thickness skin graft. The wound healed appropriately, and the patient was scheduled to begin adjuvant radiation therapy.

## Discussion

Phyllodes tumor is an uncommon fibroepithelial breast mass that accounts for less than 1 percent of female breast tumors, and typically manifests in women aged 45-49 as a painless, multinodular tumor [1]. Clinically, phyllodes tumors should be suspected when a patient presents with a palpable, rapidly growing breast mass. Although there is variability, the median size is 4 cm. Giant phyllodes tumors are those that are greater than 10 cm. As they grow larger, they can distort the contour of the breast and cause necrosis due to pressure of the overlying skin [6].

Diagnostic workup for a palpable breast mass includes mammography and ultrasound. On mammography, phyllodes tumors typically appear as smooth, multilobulated masses that can resemble other breast neoplasms, such as fibroadenomas, angiomyolipomas and other tumors, making their value limited for diagnosis of phyllodes [7]. On ultrasound, phyllodes tumors present as a hypoechoic, solid mass, well-circumscribed, with heterogeneous internal echo patterns and may contain cystic components [8]. Following diagnosis, MRI is sometimes indicated in preoperative planning, as it can be useful in evaluating the extent of the tumor and its resectability. On MRI, phyllodes tumors present as well-circumscribed masses with irregular walls and high signal intensity on T1-weighted images, and low signal intensity on T2. High tumor signal intensity on T1 can correspond to hemorrhagic infarction. Cystic changes may also be noted on MRI, with irregular walls corresponding to necrosis [9].

Definitive diagnosis of phyllodes tumors requires core biopsy and will show histological findings of leaf-like architecture, which refers to stromal overgrowth that projects into papillary protrusions reminiscent of leaf blades. Histologically, they can be categorized as benign, borderline, and malignant depending on features such as border infiltration, mitotic activity, degree of stromal cellular atypia, and stromal overgrowth [5,10].

Regarding management of phyllodes tumors, surgical excision is the standard of care. Borderline or malignant tumors usually require surgical margins greater than or equal to 1 cm to decrease recurrence rate [11]. Following mastectomy, the decision for adjuvant radiation therapy is typically based on tumor grade. Thus, for patients with borderline or malignant tumors, adjuvant therapy is recommended following surgical excision [12]. Although no standardized guidelines exist for the follow-up of malignant phyllodes tumor after resection and adjuvant therapy, most clinicians recommend clinical surveillance with semiannual imaging (such as ultrasound or mammography) during the first 2 to 3 years, followed by annual imaging for up to 5 years [13].

## Conclusion

In conclusion, phyllodes tumors are rare breast lesions. Diagnosis should be considered in patients who present with a rapidly growing breast mass. While mammography and ultrasound remain the standard workup for palpable masses, biopsy is required for definitive diagnosis. Accurate and timely diagnosis of phyllodes tumor is imperative for appropriate management. Mastectomy remains the standard of care for giant phyllodes tumors, with adjuvant therapy typically reserved for patients with malignancy.

## Author contributions

The authors declare that this is their original work, and they all approve the content of this manuscript. They confirm that this manuscript has not been published previously, in any language, in whole or in part, and is not currently under consideration elsewhere.

## Ethical clearance

This project did not involve any research and no ethical clearance was required.

## Patient consent

A written informed consent was obtained from the patient for the publication of this case report.
